# Cancer morbidity and mortality after pediatric solid organ transplantation—a nationwide register study

**DOI:** 10.1007/s00467-020-04546-y

**Published:** 2020-05-11

**Authors:** Kira Endén, Juuso Tainio, Atte Nikkilä, Ilkka Helanterä, Arno Nordin, Mikko P Pakarinen, Hannu Jalanko, Kirsi Jahnukainen, Timo Jahnukainen

**Affiliations:** 1 Department of Pediatric Nephrology and Transplantation, Children’s Hospital, University of Helsinki and Helsinki University Hospital, Helsinki, Finland; 2 Department of Pediatrics, Tampere University Hospital, Tampere, Finland; 3 Tampere University, Tampere, Finland; 4 Transplantation and Liver Surgery Helsinki University Hospital, Helsinki, Finland; 5 Pediatric Liver and Gut Research Group and Section of Pediatric Surgery, Children’s Hospital, University of Helsinki and Helsinki University Hospital, Helsinki, Finland; 6 Division of Hematology-Oncology and Stem Cell Transplantation, Children’s Hospital, University of Helsinki and Helsinki University Hospital, Helsinki, Finland

**Keywords:** Pediatric transplantation, Cancer, Adolescent, Adult, Follow-up, Mortality

## Abstract

**Background:**

The prevalence of malignancies after pediatric solid organ transplantation was evaluated in a nationwide study.

**Methods:**

All patients who had undergone kidney, liver, or heart transplantation during childhood between the years 1982 and 2015 in Finland were identified. The inclusion criteria were age under 16 years at transplantation and age over 18 years at the last follow-up day. A total of 233 (137 kidney, 53 liver, and 43 heart) transplant recipients were enrolled. Controls (*n* = 1157) matched by the year of birth, gender, and hometown were identified using the Population Register Center registry. The cancer diagnoses were searched using the Finnish Cancer Registry.

**Results:**

Altogether 26 individuals diagnosed with cancer were found, including 18 transplant recipients. Cancer was diagnosed at a median of 12.0 (IQR 7.8–17.8) years after the transplantation. The transplant recipients’ risk for cancer was significantly higher when compared with the controls (HR 14.7; 95% CI 6.4–33.9). There was no difference for different graft types. Sixty-one percent of cancers among the transplant recipients were diagnosed at age older than 18 years.

**Conclusion:**

The risk for cancer is significantly higher among young adults having undergone solid organ transplantation during childhood in comparison with population controls. Careful follow-up and attention to prevent cancers throughout adulthood are warranted.

**Electronic supplementary material:**

The online version of this article (10.1007/s00467-020-04546-y) contains supplementary material, which is available to authorized users.

## Introduction

Solid organ transplantation is the only curative treatment for end-state organ failure. Successful pediatric kidney (KTx), liver (LTx), and heart (HTx) transplantations have been performed for more than three decades, and a new patient group, long-term survivors after pediatric solid organ transplantation, has emerged. The knowledge about late complications as well as morbidity and mortality after solid organ transplantation has increased in recent years. The most common long-term complications are related to cardiovascular disease, infections, and malignancies [1–5].

The risk for cancer is significantly increased in all transplant recipients, and especially among patients with a history of pediatric solid organ transplantation [6–10]. The incidence of de novo malignancies among solid organ recipients varies between 2 and 23%, depending on study population [6–8, 10–13], and the risk for different cancers has been reported to be as much as 30-fold higher than in the non-transplant population [10]. The increased prevalence of cancers is related to exposure to immunosuppressive medication and oncogenic viruses [14, 15].

The most common cancer after pediatric solid organ transplantation is post-transplant lymphoproliferative disorder (PTLD), which altogether covers 52–80% of all cancers after solid organ transplantation [6, 7, 10–12, 16]. According to the 2016 WHO update, PTLD was classified as lymphoid neoplasms into six categories: plasmacytic hyperplasia, infectious mononucleosis, florid follicular hyperplasia, and polymorphic, monomorphic, and classical Hodgkin lymphoma [17]. Previous studies have shown that the incidence of PTLD is highest after intestinal, liver, heart, and lung transplantation [6, 7]. In addition to the level of immunosuppression, young age at transplantation and Epstein-Barr virus (EBV) seronegativity are risk factors for PTLD [7, 8].

It has also been shown that PTLD appears earlier after transplantation when compared with other malignancies [8]. Kitchlu et al. [10] reported recently that 23% of all deaths among transplant recipients were caused by malignancies and that the majority of cancers (68%) leading to death were PTLDs. In the study by Serrano et al. [18] among pediatric KTx recipients, 64% of deaths were caused by cancer. A recent report from the USA showed that transplant recipients had an increased risk for cancer mortality even in comparison with other cancer patients [19].

In most of the previous studies, the follow-up time was relatively short and the data on cancer morbidity and mortality in adulthood after pediatric transplantation are scarce. The goal of this national registry-based study was to evaluate the cumulative cancer incidence and cancer-associated long-term mortality in young adults with a history of kidney, liver, or heart transplantation during childhood compared with date of birth, gender, and area of residence-matched controls who were identified for each transplant case from the Finnish Population Register Center. We hypothesized that the cumulative cancer incidence is increased among transplant recipients.

## Materials and methods

### Ethics

The Ethics Committee of Helsinki University Hospital, the Institute for National Health and Welfare, and the Office of the Data Protection Ombudsman approved the study protocol.

### Study population

All pediatric solid organ transplantations in Finland have been performed at the Helsinki University Hospital since 1982. We identified all the pediatric kidney, liver, and heart transplant recipients transplanted between January 1, 1982 and December 31, 2015. The inclusion criteria for the study were age under 16 years at transplantation and age over 18 years at the last follow-up day, December 31, 2015. Those transplant recipients whose age at the last follow-up day would have been over 18 years but who had died were included in the study population. Patients with a cancer diagnosis before transplantation (*n* = 13) were excluded from the analyses.

A total of 233 (137 kidney, 53 liver, and 43 heart) transplant recipients were included in the study, and each transplant recipient had 3–5 years of birth-, gender-, and hometown-matched controls. Hometown matching was based on residence at moment of transplantation. Totally 1157 controls were identified using the Population Register Center registry. The presence of chronic diseases was evaluated from the Specially Reimbursement Drug Registry. Nine controls had connective tissue disease, mainly rheumatoid arthritis, and ten controls had colitis ulcerosa. One of these control subjects had cancer. One control had kidney transplant during adulthood and was excluded from the study population. By linkage to the Finnish Cancer Registry, all cancer diagnoses among the transplant recipients and the control subjects diagnosed between 1982 and 2015 were searched. The coverage of the Finnish Cancer Registry is 100%, because in Finland, it is obligatory by the law to report all diagnosed cancers to the registry. Classification of disease for oncology (ICD-O-3) was used to categorize the diagnoses [20]. We also reviewed the medical records of all the patients with reported cancer in order to verify the accuracy of the registry data as well as to obtain information about the immunosuppressive medication and EBV status at the time of cancer diagnosis.

### Immunosuppression protocol

Anti-thymocyte globulin (ATG) has been used as induction therapy in HTx patients from the beginning of our transplant program. Until the year 2000, kidney and liver recipients did not receive either monoclonal or polyclonal antibodies as induction therapy. Since the year 2000, basiliximab has been used as induction therapy in kidney and liver recipients. The maintenance immunosuppression protocol of the transplant recipients consisted of triple medication. The most commonly used drug combination after KTx and LTx was cyclosporine A (CsA), azathioprine (AZA), and methylprednisolone. In HTx recipients, a combination of CsA and AZA was used as primary immunosuppression until around the year 2010, after which tacrolimus with AZA or mycophenolic acid (MPA) has been the first-choice immunosuppression. Methylprednisolone was initially dosed daily and later switched to alternate-day dosing at 3–6 months after transplantation.

All the transplant recipients visit our institution at least annually. These follow-up visits include e.g., evaluation of graft function, chest X-ray in HTx recipients, abdominal ultrasonography in KTx and LTx recipients, and screening for EBV nucleic acid testing.

### Statistical analyses

SPSS statistics 24 (SPSS Inc., Chicago, IL, USA) and R version 3.4.4 were used for data analyses. Data between two subject groups were compared using Kruskal-Wallis test and Mann-Whitney *U* test for continuous variables, and Pearson chi-squared test and Fisher’s exact test for categorical variables. The reported survival analyses were carried out with Cox proportional-hazards models and the PH assumption was evaluated with Schoenfeld residuals. Cumulative incidence plots and forest plots were used as visualization aids. A *p* value less than 0.05 was considered statistically significant and all tests were two-tailed. No corrections for multiple testing were used. Cumulative survival was evaluated with Kaplan-Maier estimator. The event was defined as a death from any cause.

## Results

### Patient characteristics

The descriptive characteristics of the study subjects are shown in Table 1. The primary causes for kidney transplantation included congenital nephrotic syndrome of the Finnish type (34%), congenital anomalies of kidneys or urinary tract (23%), cystic diseases (18%), glomerulonephritis (13%), and miscellaneous (12%) diagnoses; for liver transplantation: biliary atresia (42%), metabolic diseases (30%), acute liver failure (15%), and miscellaneous (13%); and for heart transplantation: congenital heart defect (49%) and cardiomyopathies (51%). The median age of all the transplant recipients at the time of the study was 24.6 (range 0.8–44.0) years and for those alive at the last follow-up day 25.8 (18.3–44.0) years. The median follow-up time of all the recipients was 18.0 (0.3–30.0) years. In total, sixteen kidney and ten liver transplant recipients received a re-transplant. The mortality rate was 25.8% among the transplant recipients and 0.2% among the controls (*p* < 0.001).

**Table 1:** Clinical characteristics of kidney transplant (KTx), liver transplant (LTx), and heart transplant (HTx) patients and controls

	All Tx *n* = 233	KTx *n* = 137	LTx *n* = 53	HTx *n =* 43	Controls *n =* 1157	*p* value
Age at time of study (alive) (years)	25.8 (18.3–44.0)	26.3 (18.4–44.0)	25.7 (19.7–37.2)	24.4 (18.3–38.4)	26.4 (18.1–44.1)	0.26
Post-Tx time (years)	18.0 (0.3–30.0)	20.0 (0.7–30.0)	15.0 (0.4–27.0)	13.0 (0.3–25.0)		
Males, *n* (%)	139 (59.7)	92 (67.2)	25 (47.2)	22 (51.2)	691 (59.7)	0.52
Age at time of Tx (years)	7.9 (0.4–15.9)	7.9 (1.1–15.9)	4.9 (0.4–15.9)	10.3 (1.0–15.9)		
Malignancy, *n* (%)	18 (7.7)	14 (10.2)	2 (3.8)	2 (4.7)	8 (0.7)	< 0.001*
Alive, *n* (%)	173 (74.2)	117 (85.4)	30 (56.6)	26 (60.5)	1155 (99.8)	< 0.001*
Age of cancer diagnosis (years)	18.9 (3.3–33.9)	18.7 (4.1–25.6)	18.6 (3.3–33.9)	17.3 (12.2–22.3)	26.2 (13.0–29.3)	0.13
Time from Tx to cancer diagnosis (years)	12.0 (1.8–23.6)	13.3 (6.9–23.6)	10.7 (1.8–19.7)	7.9 (4.7–11.1)		

Data are presented as median (range) or number of subjects (%). *p* value between all Tx recipients and controls. *p* values from the Mann-Whitney *U* test and from Fischer’s exact test, as appropriate

*Tx* transplantation

*Statistically significant

### Malignancies

**Fig. 1:**
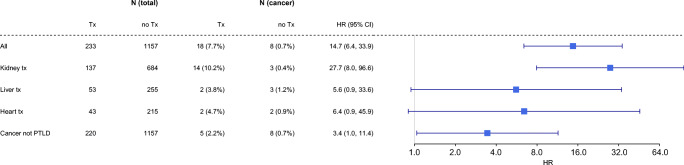
Difference in cancer risk between transplant recipients and controls. *HR* hazard ratio, *95% CI* 95% confidence interval, *Tx* transplant

**Fig. 2:**
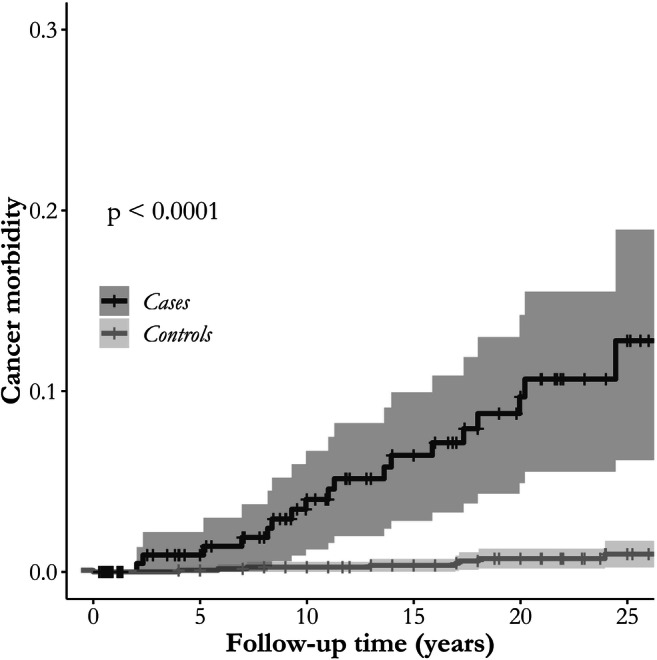
The cumulative cancer incidence among transplant recipients and matched controls during follow-up period by Cox proportional-hazards models

Altogether 26 cancers were found: 18 in the transplant recipient group and eight among the controls (Table 2). The transplant recipients’ HR for cancer diagnosis was 15-fold higher than the controls’ (95% CI 6.4–33.9) (Fig. 1)—additional data are given in Online Resource (ESM_1). The cumulative cancer incidence was 0.95% during the first 5 years post-transplantation after which it gradually increased up to 12.11% during the follow-up period (up to 25 years) (Fig. 2). At the time of cancer diagnosis, the transplant recipients were nearly 10 years younger when compared with the controls (median 18.7 IQR 14.1–22.8 vs. 26.2 IQR 17.2–28.6 years); however, the difference was not statistically significant (*p* = 0.129).

**Table 2:** Cancer diagnoses in pediatric transplant recipients and their respective controls

	All Tx *n =* 233	Controls *n =* 1157	KTx *n =* 137	Controls *n =* 684	LTx *n =* 53	Controls *n =* 258	HTx *n =* 43	Controls *n =* 215
Lymphoma	15	1	12	1	1		2	
NHL	12		10				2	
HL	1	1	1	1				
Small B cell	1				1			
Unknown*	1		1					
Genitouretral	1	3	1	1		2		
Skin	2		1		1			
Other		4		1		1		2
TOTAL	18	8	14	3	2	3	2	2

*Tx* transplant, *KTx* kidney transplant, *LTx* liver transplant, *HTx* heart transplant, *NHL* non-Hodgkin lymphoma, *HL* Hodgkin lymphoma

Other- appendix carcinoma, thyroid gland adenoma, breast carcinoma, osteosarcoma

*Classified in PTLD

In the transplant group, all tumors were cancers, whereas in the control group, one tumor was classified as borderline malignant tumor (mucinous cystic tumor in the ovary (Table 2). PTLD was the most common cancer diagnosis among the transplant recipients, accounting for 78% of all tumor types in this group. Among the controls, genitourinary cancer was the most frequent tumor type (Table 2).

One recipient and one control subject had two separate cancers diagnosed. One female liver transplant recipient had a small B cell lymphoma at the age of 3 years, and 23 years later, a large B cell lymphoma in the ileum, which led to her death. In the control group, one male had a Hodgkin lymphoma at the age of 13 and 10 years later, a basal cell carcinoma of the skin. Only the first malignancy of each study subject was included to the study.

All the control subjects diagnosed with cancer were alive at the time of the study, while in the transplant group, nine (50%) of the 18 patients with cancer had died. Twelve percent (7/60) of all deaths among transplant patients were due to cancer. The highest rate of death caused by malignancy was in the KTx group, where 25% of all deaths were cancer-related. Among LTx and HTx recipients, the cancer-related death rate was 4 and 6 %, respectively. All but one of the deaths caused by cancer occurred in patients with PTLD. In the transplant group, the five-year survival after cancer diagnosis was 69% (95% CI 0.50–0.96).

### Cancer among the transplant population

**Fig. 3:**
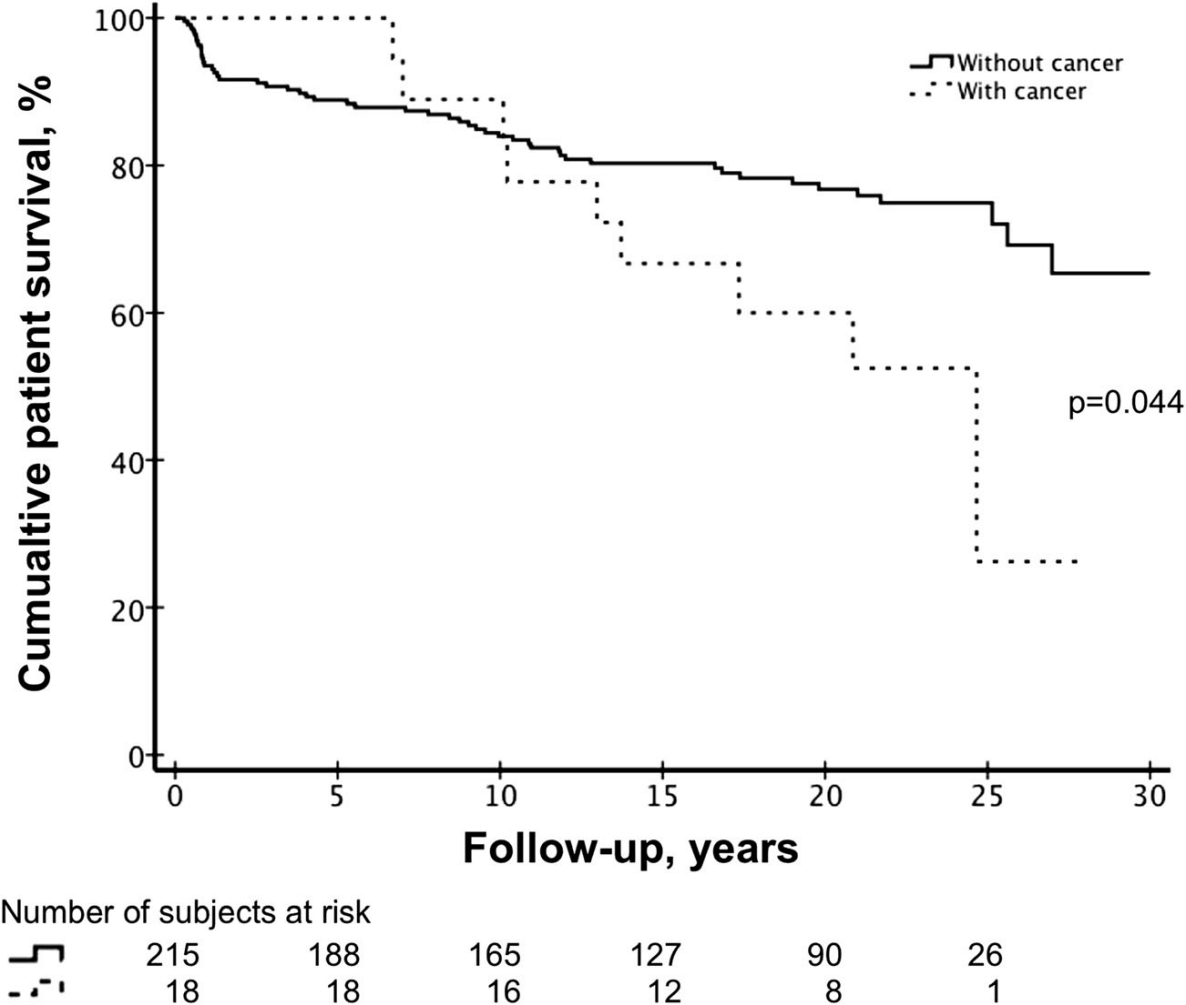
Kaplan-Meier survival curve for transplant recipients with and without cancer diagnosis

The patient demographics did not differ significantly between the transplant recipients with or without cancer diagnosis (Table 3). The recipients with cancer diagnosis tended to be younger at the time of transplantation than those without cancer diagnosis (median 5.9 IQR 1.7–10.6 vs. 8.1 IQR 2.6–13.4 years, respectively); however, the difference did not reach statistical significance (*p* = 0.192). At the time of the study, 76% of the non-cancer transplant recipients were alive, while only 50% of the recipients with cancer had survived (Fig. 3). The time from cancer diagnosis to death was remarkably short, median 0.66 years (IQR 0.08–1.61) and 0.46 years (IQR 0.08–0.99) in all cancer patients and in the patients with PTLD, respectively. Among re-transplanted recipients, no malignancies were reported.

**Table 3:** Clinical characteristics of transplant recipients with and without cancer

	With cancer *n =* 18	Without cancer *n =* 215	*p* value
Age at time of Tx (years)	5.9 (1.1–14.3)	8.1 (0.4–15.9)	0.192
Males, *n* (% within the group)	11 (61.1)	128 (59.5)	0.553
Post-Tx time (years)	18.7 (6.7–28.0)	18.0 (0.3–30.0)	0.849
Age at index day* (years)	24.3 (11.6–36.0)	24.7 (0.8–44.0)	0.469
Alive, *n* (% within the group)	9 (50.0)	164 (76.3)	0.023§
Age at death (years)	20.2 (11.6–25.9)	16.6 (0.8–37.5)	0.168

Data are presented as median (range) or number of subjects (%). *p* values from the Mann-Whitney *U* test and from Fischer’s exact test, as appropriate

*Tx* transplantation

*Index day, day of death, or last follow-up day

§Statistically significant

**Fig. 4:**
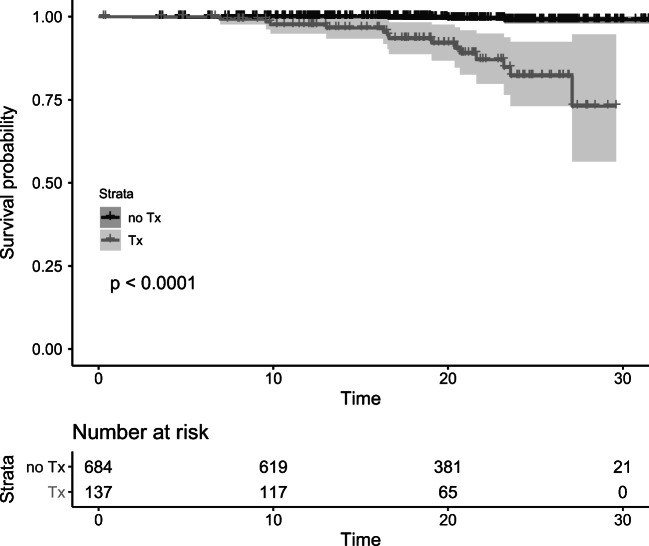
Kidney transplant recipients’ cancer risk compering to controls. Tx-transplantation

The kidney transplant recipients had a higher risk for cancer (HR 27.7, 95% Cl 8.0-96.6) compared with controls (Fig. 4). The vast majority of all cancers after kidney transplantation (83%) were PTLDs (Table 2). Among the liver and heart transplant recipients, the risk for cancer tended to be increased when compared with controls (HR 5.6, 95% CI 0.9–33.6; and HR 6.4, 95% CI 0.9–45.9, respectively) (Fig. 1); additional data are given in Online Resources (ESM_2, ESM_3). The liver transplant recipients were younger at the time of transplantation than other recipients, and the kidney transplant recipients had the longest follow-up time. Median age at cancer diagnosis did not differ between the transplant groups (Table 1). Multivariate analysis with Cox proportional-hazards models (Table 4) showed that type of transplant (liver HR 0.50 (95% CI 0.11–2.24), heart HR 0.63 (95% CI 0.14-–.87)), age at time of transplantation (5–9.99 years HR 0.94 (95% CI 0.25–3.55), over 10 years HR 0.83 (95% CI 0.27–2.55)), year of transplant (1994–2003 HR 1.27 (95% CI 0.46–3.49), 2004–2015 HR 1.56 (95% CI 0.16–15.9)), or sex (female HR 0.92 (95% CI 0.35–2.42)) did not influence the cancer risk.

**Table 4:** Potential and known risk factors for cancer after pediatric solid organ transplantation with univariate and multivariate Cox proportional-hazards models

	Univariate		Multivariate	
	HR	95% CI	HR	95% CI
Organ				
Kidney	Ref.		Ref.	
Liver	0.50	0.11, 2.19	0.50	0.11, 2.24
Heart	0.63	0.14, 2.79	0.63	0.14, 2.87
Tx age (years)				
0–4.99	Ref.		Ref.	
5–9.99	0.99	0.27, 3.68	0.94	0.25, 3.55
10–15.99	0.86	0.30, 2.43	0.83	0.27, 2.55
Tx year				
1982–1993	Ref.		Ref.	
1994–2003	1.26	0.47, 3.36	1.27	0.46, 3.49
2004–2015	1.38	0.16, 12.2	1.56	0.16, 15.9
Sex				
Male	Ref.		Ref.	
Female	0.98	0.27, 3.68	0.92	0.35, 2.42

For categorical variables, the reference category is marked with “Ref.”

*HR* hazard ratio, *95% CI* 95% confidence interval, *Tx* transplant

Eleven (61%) of all cancers among the transplant recipients were diagnosed after their 18th birthday while the remaining seven (39%) cancers were detected in patients under 18 years of age. The age at the time of transplantation did not differ significantly between the patients with cancer diagnosed before or after 18 years of age (2.4 years, IQR 1.5–8.8 vs. 8.4 years, IQR 2.3–11.3, *p* = 0.179) and, consequently, the time between transplantation and cancer diagnosis was significantly shorter among the younger age group (7.1 years, IQR 4.0–10.6 vs. 15.2 years, IQR 10.5–19.7 *p* = 0.007). In both age groups, PTLD was the most common cancer type, accounting for 71% among patients under 18 years at time of cancer diagnosis and 82% in the age group older than 18 years at cancer diagnosis. Additionally, one squamous cell carcinoma and one small B cell lymphoma were found in the younger age group, and one testicular teratocarcinoma and one basal cell carcinoma of the skin in recipients older than 18 years.

### PTLD, EBV serology, and type of immunosuppression

A total of 14 Tx recipients had PTLD. The median age at the time of transplantation or the time between transplantation and cancer diagnosis was not significantly different (*p* = 0.382) between recipients with PTLD (median 4.2 (IQR 1.7–10.5) years) and other types of cancer (median 9.7 (IQR 3.3–13.3 years). The median time from transplantation to the cancer diagnosis was 12.9 (IQR 9.2–16.9) years in the PTLD group and 7.6 (IQR 3.1–16.7) years in the patients with other cancers (*p* = 0.245). Most of the recipients with cancer other than PTLD were alive at index day (75%) while 43% of the patients in the PTLD group were alive (*p* = 0.576).

In all but one patient with PTLD, EBV serology was available and positive at the time of PTLD diagnosis as a sign of previous or current EBV infection. In the remaining case, EBV serology was not available. Pre-transplant EBV serology was available for seven patients and all but one had seroconversion between transplantation and PTLD diagnosis. In ten cases, histological examination of the tumor tissue was available. EBER in situ hybridization (ISH) was performed where possible (*n* = 9) and EBER positivity was found in four cases at the time of PTLD diagnosis. Blood EBV viral load was available from seven patients and it varied between 8400 and 225,200 copies/mL. In two cases, the information about immunosuppression at time of PTLD was not available. Concerning calcineurin inhibitors, 9 patients were treated by CsA and 3 by tacrolimus. Seven patients were on MPA, four on AZA, and one patient receive none of these.

## Discussion

Cancer is a known threat after solid organ transplantation [7, 10, 19]. The increased cancer risk is mainly due to prolonged exposure to immunosuppressive medication, and pediatric transplant recipients are therefore considered a high-risk group [21, 22]. The majority of previous reports describe early-appearing cancers diagnosed shortly after pediatric transplantation, whereas studies on adult patients with a history of pediatric solid organ transplantation are scarce [8, 13, 18]. In the present study, based on the data from the National Population Register Center and the Finnish Cancer Registry, we focused on adult survivors after pediatric transplantation. In our cohort, 61 % of cancers were detected after the subjects’ 18th birthday. The novelty of this study is the long follow-up time after pediatric solid organ transplantation.

In the present study, cancer was diagnosed in 8 % of the solid organ transplant recipients after a median follow-up time of 18.0 years, which is in accordance with the previous data reported from pediatric and adult cohorts [8, 10, 23, 24]. According to Simard et al. [8], the cumulative incidence of cancer in pediatric population is 7% with a median follow-up time of 9.5 years, while Kitchlu et al. have recently reported a cumulative incidence of 20% during 10.8 years’ follow-up [10].

The vast majority of cancers in pediatric reports are diagnosed within the first few years after transplantation [7, 10], which is probably due to more intensive immunosuppression regimen and higher risk for cancers related to viral infections, such as PTLD. Among the adult recipients, cancer incidence increases further along the time from transplantation [24], which is at least partly explained by the higher incidence of cancer at older age in general. In the present study, the majority of cancers were diagnosed at young adult age, and a study by Park et al. [25] showed that highest risk for post-transplant malignancy was among transplant age group 0–19 years.

The current study population differs from most of the previous pediatric studies because the patient selection was restricted to pediatric recipients older than 18 years of age at the time of the study, and we had three different solid organ transplantation groups. This gives a novel option to show that a significant age-dependent increase in cumulative cancer incidence is detected in early adulthood in pediatric transplant population after a median follow-up time of 18 years, and compares kidney, liver, and heart transplant recipients’ risk of cancer. The difference in cumulative cancer incidence and cancer-specific mortality was significantly higher when compared with matched controls, which is in accordance with the previous registry studies by Benoni et al. [26] and Acuna [27]. However, in the present study, the cancer risk and cancer-related mortality were lower among the liver and heart transplant recipients than the kidney transplant recipients, which was a somewhat surprising finding. Studies comparing cancer risk in different transplant groups are scarce. In two large registry studies, heart and liver transplant recipient’s risk for malignancies was higher than kidney transplant recipients [7, 10]. In our study population, kidney transplant recipients had the longest follow-up time, and the patients were younger at the time of transplantation than heart transplant recipients.

Our findings are compatible with Serrano et al. [18] in that the difference in cumulative cancer incidence between transplant recipients and matched controls increases with longer follow-up time and does not show a plateau during 25 years of follow-up (Fig. 2).

In our transplant cohort, the most common malignancy was PTLD (78%), which is in accordance with the previous data showing that PTLD and lymphomas are the most frequently found malignancies among transplant recipients [7, 10, 12, 18]. Unlike previous studies [16, 28–30], our series did not include any early-onset PTLDs (diagnosed within 2 years after transplantation). The median post-transplantation time to cancer diagnosis was 12.9 years among the PTLD patients and 12.0 years in patients with other types of cancers, which is longer than in the studies by Smith and Koukourgianni, [9, 11] but comparable with a study among young KTx recipients where the median time to cancer was 14.7 years [31]. In a Swedish registry study, all cancer cases except non-Hodgkin lymphoma occurred during adulthood [8]. This confirms the present finding that a significant number of cancers after pediatric transplantation appear during adult age. PTLD recurrences are reportedly rare. In our cohort, only one patient with a history of small B cell lymphoma during childhood developed non-Hodgkin lymphoma over 20 years after the primary lymphoma diagnosis. In earlier studies, the reported PTLD relapse rate varies between 0 and 12% [32–34]. The risk for PTLD recurrence was found to be higher in patients on steroids after PTLD diagnosis [35] and lower in patients treated with rituximab and low-dose chemotherapy [34].

In our cohort, the incidence of solid tumors other than PTLD and non-melanoma skin cancers was lower than those reported in earlier studies [7–9, 13, 18]. The lower incidence of solid tumors may be at least partly explained by population and environmental differences in cancer risk. In the Nordic countries, the level of ultraviolet light exposure is low, and transplant recipients are well-informed to avoid unnecessary exposure to sunlight and to use sun protection. In one Dutch study, cancer incidence rate during 30-years follow-up was relatively high, 41% [13]. Like in the report from Australia and New Zealand, the majority of cancers were non-melanoma skin cancers [31]. Data from a long-term follow-up study on Nordic pediatric liver transplant patients showed that age is a significant risk factor for cancer, and the absolute risk for most cancers increases in patients older than 20 years of age [36].

Furthermore, remarkably high mortality to post-transplant malignancies has been reported by earlier studies [18, 19, 26, 27]. Lower survival has been linked to PTLD diagnosis and age over 18 years at the time of cancer diagnosis. In the present study, the percentage of the patients who died of cancer was 12%, which is in accordance with the study by Ploos et al. [13]. The mortality rate reported from Minnesota was remarkable high, at 64% [18]. The Minnesota study presented data from an earlier era, which at least partly explains the higher mortality rate. Also in the present study, the highest numbers of cancer-caused deaths were among KTx recipients. LTx and HTx recipients’ cancer mortality was comparable and lower than KTx recipients’. In the present study, the time interval from cancer diagnosis to death was relatively short, which supports the previous data. This is probably caused by aggressive progression of post-transplant cancers, especially PTLD, and increased toxicity of cancer treatments in patients with a history of solid organ transplantation and chronic disease. Based on these findings, careful follow-up by health care providers and self-monitoring for cancer symptoms in adults with a history of pediatric solid organ transplantation is warranted.

A difference in the cancer incidence between different graft types was observed, but was not statistically significant. Yanik et al. [7] have recently shown in a register-based data set consisting of more than 17,000 patients that the incidence of malignancies is significantly increased among pediatric solid organ transplant recipients, with the highest incidence in small bowel recipients, followed by heart/lung, liver, and kidney transplant patients. The vast majority of the diagnosed malignancies were early onset non-Hodgkin lymphomas, and the highest risk for cancer was among patients less than 12 months from transplantation. Eighteen percent of the malignancies were diagnosed in patients aged 18 years or more. Kitchlu et al. [10] have recently shown that pediatric liver and heart transplantation recipients have the highest cancer incidence. In the present study, the highest cancer incidence was among kidney transplant recipients, and the difference compared with matched controls was statistically significant only in this patient group. This finding may be due to unequal sample sizes, longer follow-up time in kidney transplant recipients than in other recipients, and the overall higher early mortality among liver and heart recipients than among kidney recipients.

Because this is a register-based follow-up study, our possibilities to analyze risk factors affecting cancer incidence are somewhat restricted. Surprisingly, in our cohort, the cancer risk was not affected by the transplant era. On the contrary, a slightly higher hazard ratio for cancer diagnosis was seen in recipients transplanted during the twenty-first century compared with those transplanted during the earlier decades. This may be due to changes in immunosuppression protocol. CsA- and AZA-based immunosuppression was used in the earliest transplant cohort (1982–1993) in our study, and the use of tacrolimus and mycophenolate has increased after that. On the other hand, CsA was the most-used calcineurin inhibitor (64%), and MPA was used in 44% of the patients with cancer diagnosis. Another difference from the early era at our institution is that induction therapy with basiliximab has been used in kidney and liver transplant recipients since the year 2000.

The main weakness of the present study is the relatively small number of study subjects. On the other hand, this is a nationwide study with a 100 % coverage of both transplant recipients and cancer diagnoses. The follow-up time exceeds with that in the majority of previous studies. In addition, the control group consists of population-based age-, gender-, and hometown-matched subjects, which in our opinion minimizes the regional differences in cancer incidence.

In conclusion, improved graft and patient survival after pediatric solid organ transplantation have raised new challenges, such as long-term effects related to the primary disease and life-long exposure to immunosuppression. It is likely that immunosuppression increases the risk for malignancies, especially those triggered by viral infections. Recommendations for cancer screening after solid organ transplantation have been made [37], but the recommendations vary and have mostly been made for kidney transplant recipients. Based on our present findings, pediatric kidney, liver, and heart transplant recipients have elevated risk for cancer morbidity, which increases further beyond the third and fourth decades of life. This necessitates an active, systematic, and coherent screening schedule for surveillance.

## Electronic supplementary material

ESM 1 Transplant recipients’ cancer risk compering to controls (PDF 48 kb)

ESM 2 Liver transplant recipients’ cancer risk compering to controls (PDF 44 kb)

ESM 3 Heart transplant recipients’ cancer risk compering to controls (PDF 47 kb)
