# Supplementary material for: Cancer morbidity and mortality after pediatric solid organ transplantation—a nationwide register study
Source: Pediatr Nephrol. 2020 May 11;35(9):1719–28. doi: 10.1007/s00467-020-04546-y (PMC7385020; doi:10.1007/s00467-020-04546-y)
Supplement: Supplementary file 1 — Transplant recipients’ cancer risk compering to controls (PDF 48 kb) [file 467_2020_4546_MOESM1_ESM.pdf]

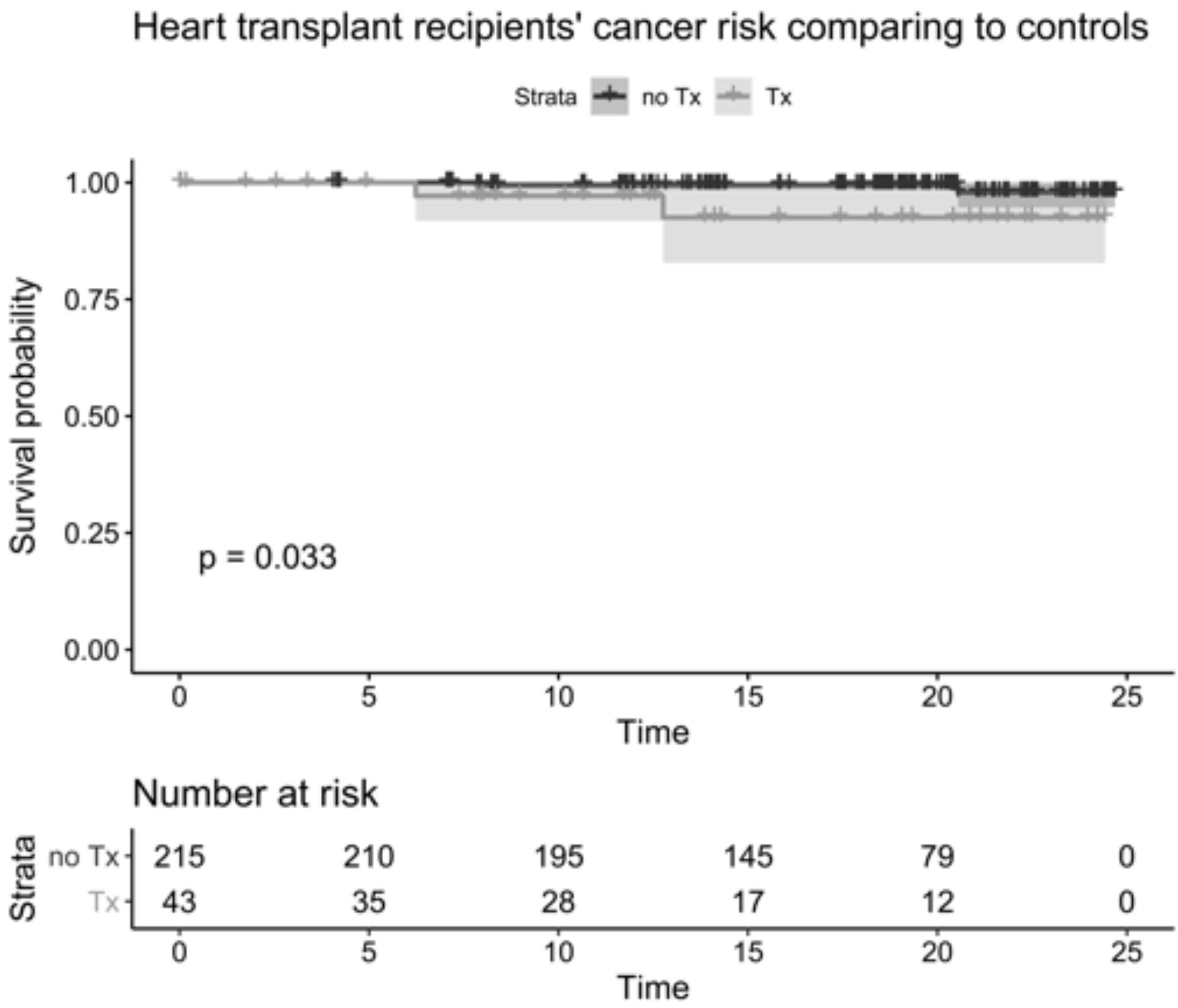

Cancer morbidity and mortality after pediatric solid organ transplantation – a nationwide register study, Pediatric Nephrology,
Kira Endén, Juuso Tainio, Atte Nikkilä, Ilkka Helanterä, Arno Nordin, Mikko P Pakarinen, Hannu Jalanko, Kirsi Jahnukainen,
Timo Jahnukainen, Helsinki University, kira.enden@helsinki.fi
